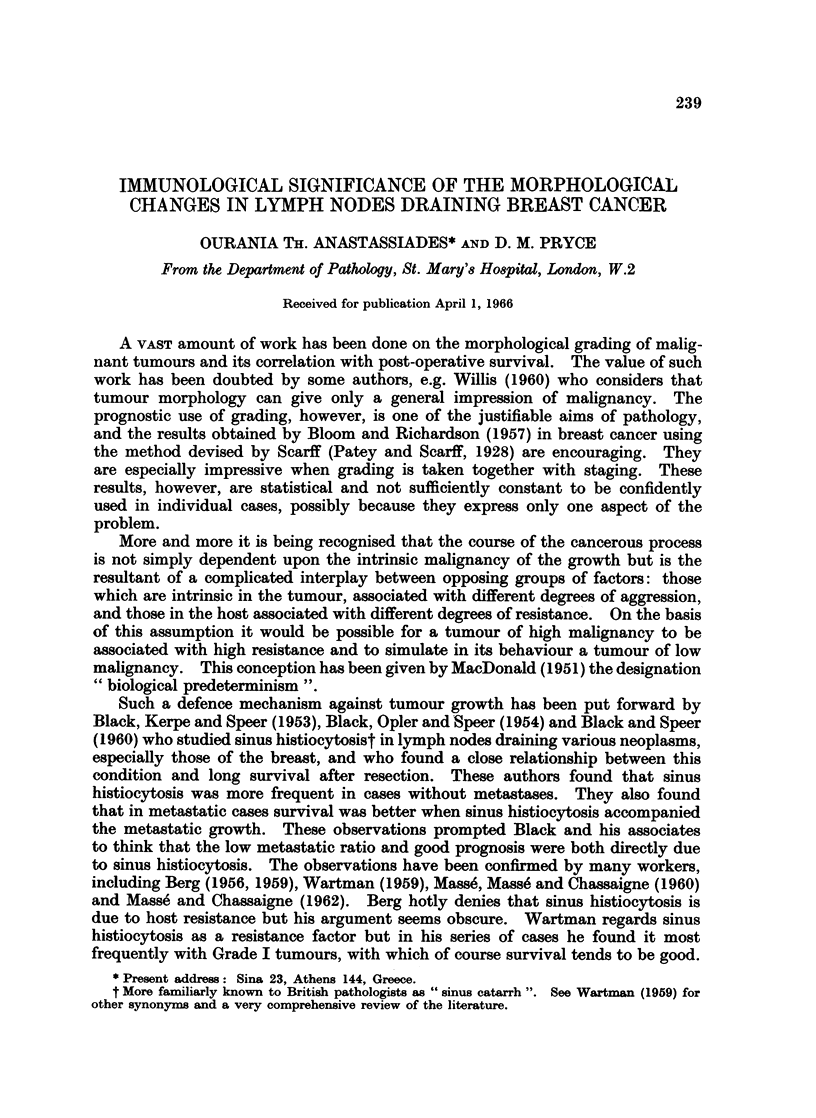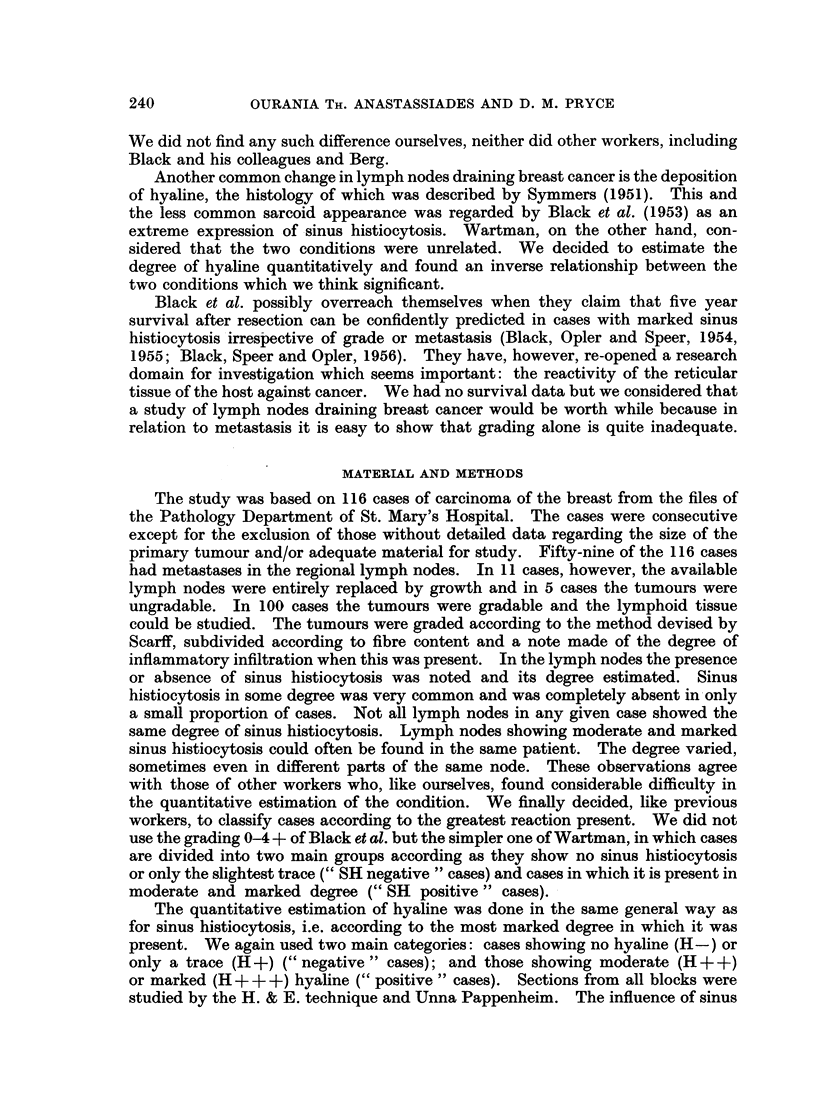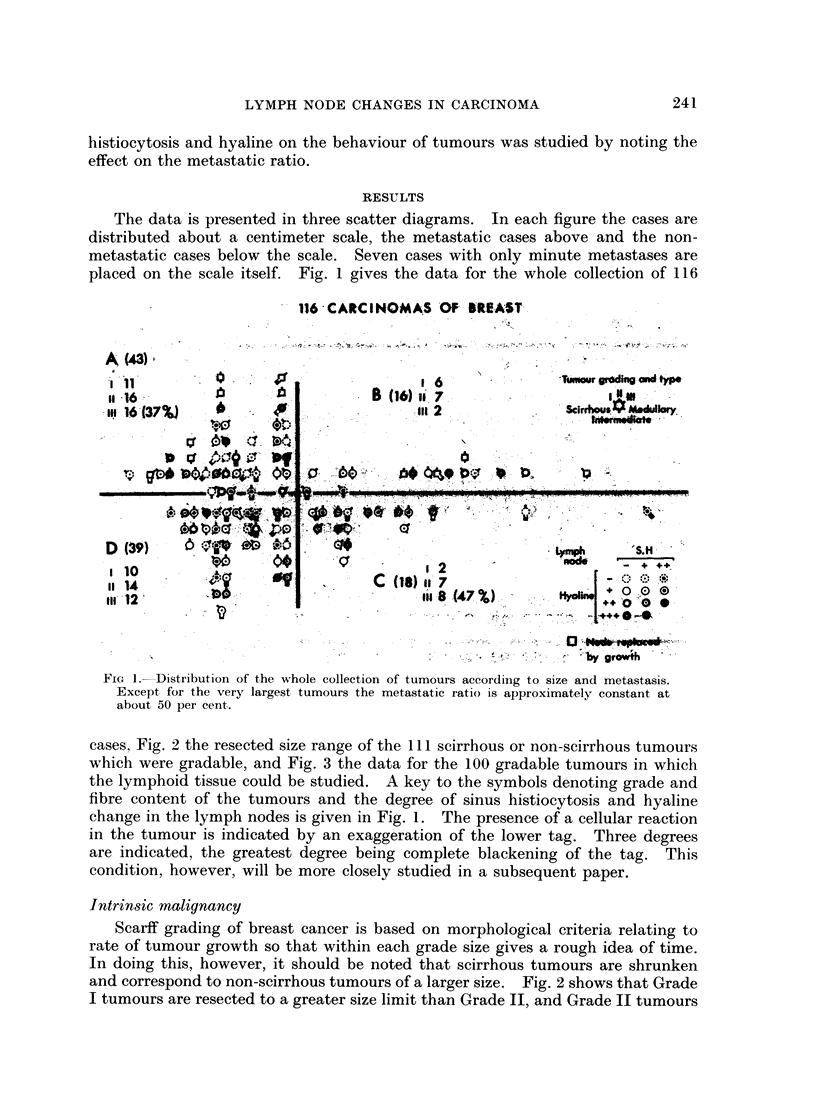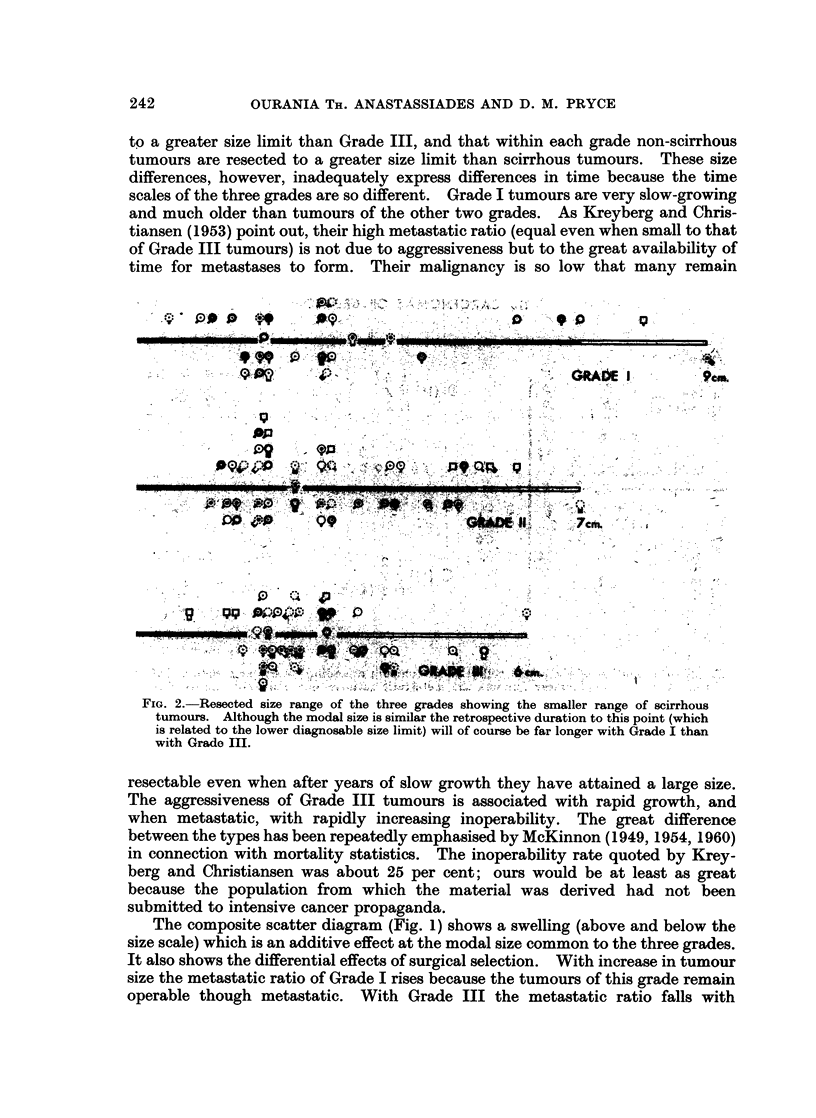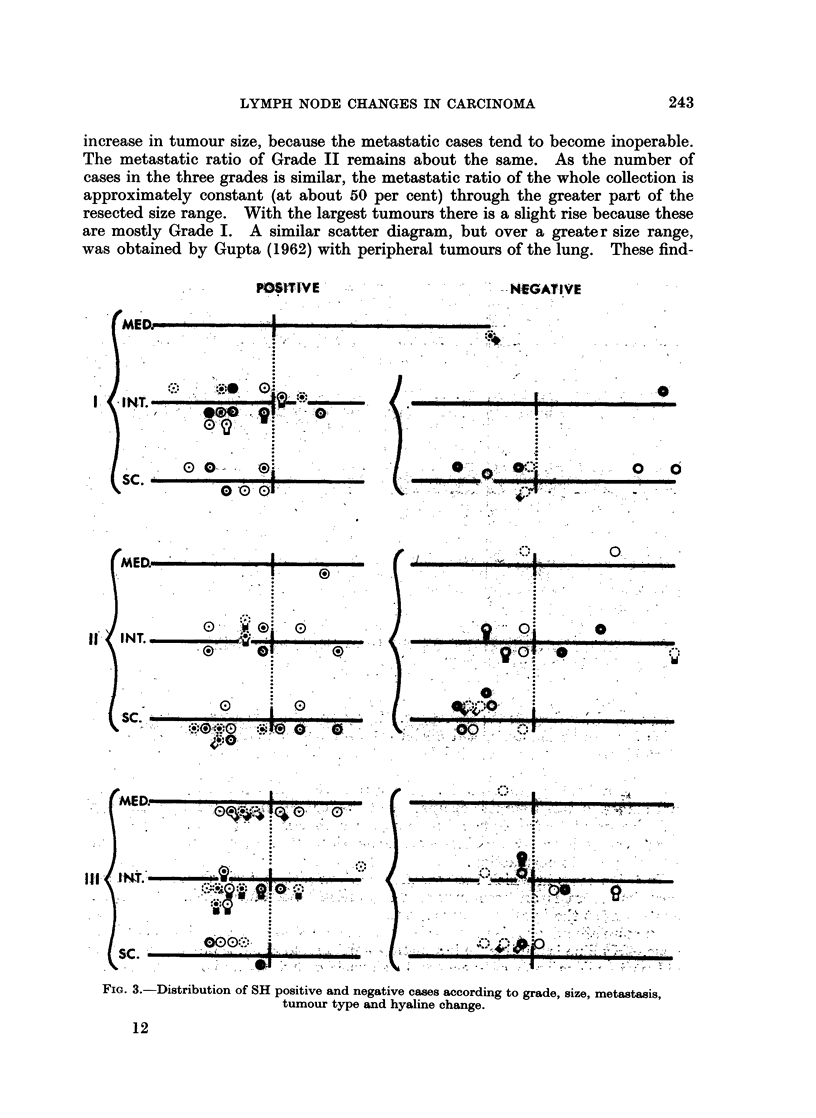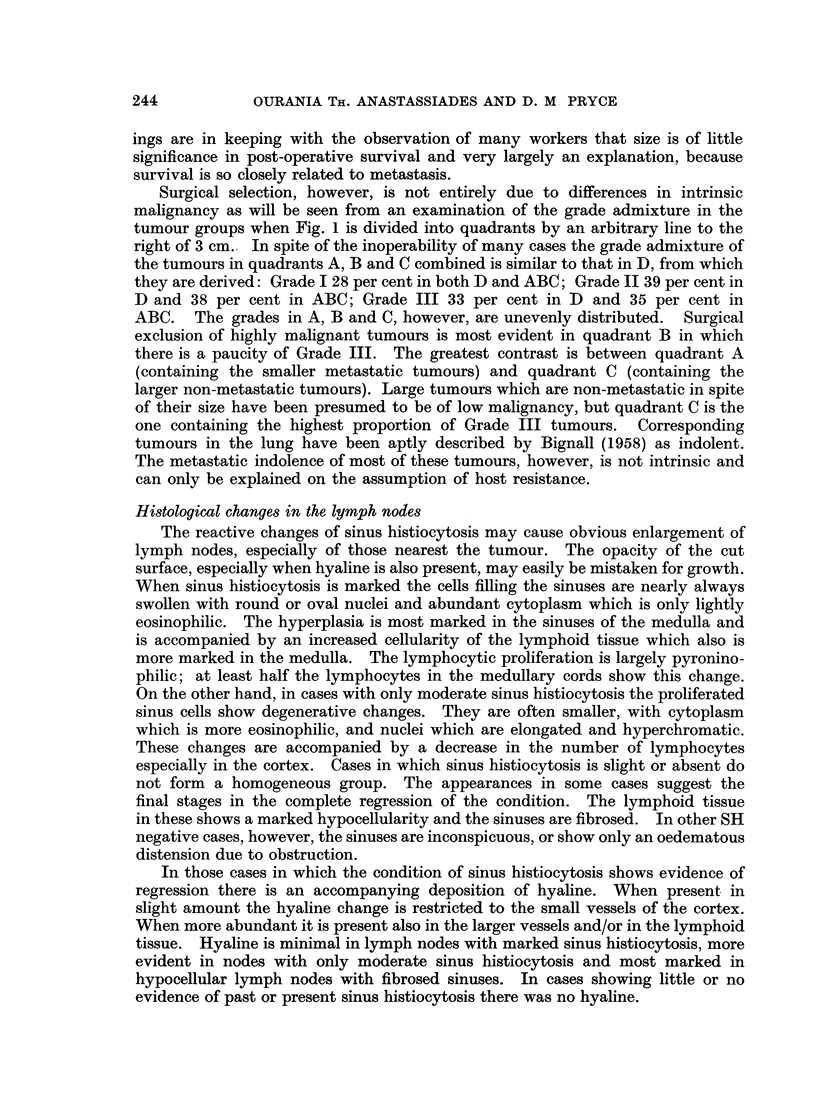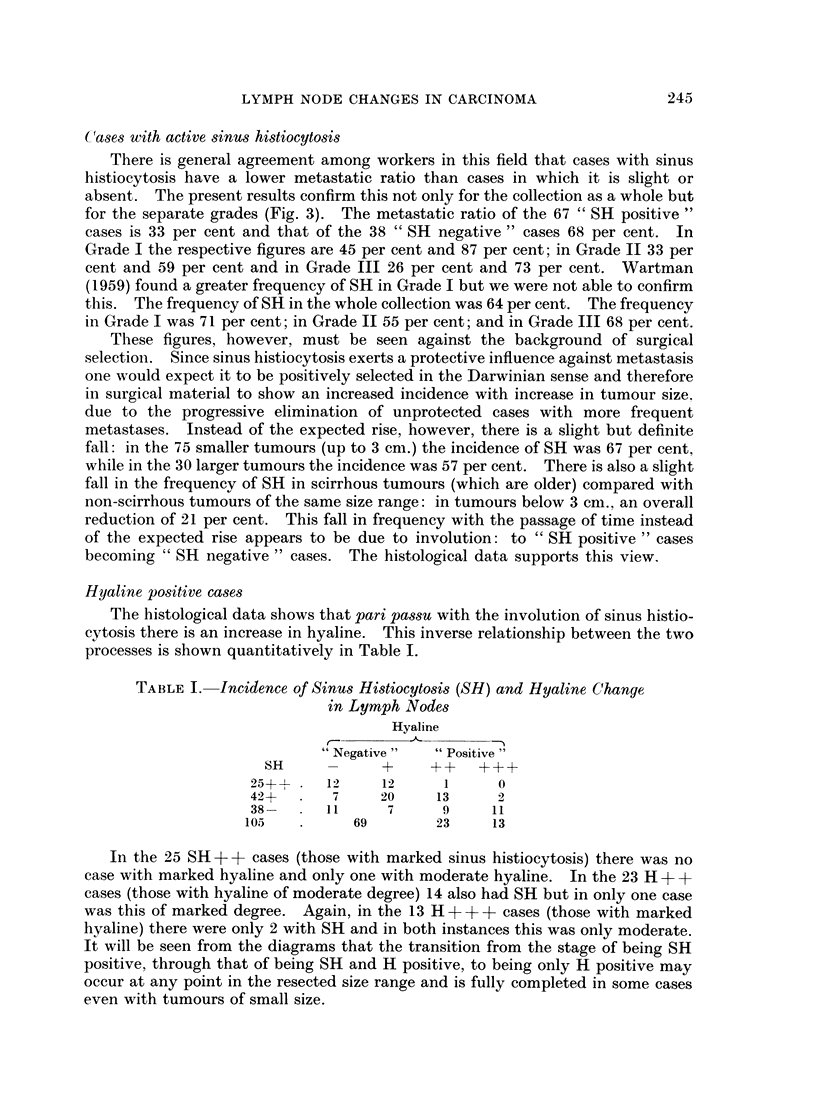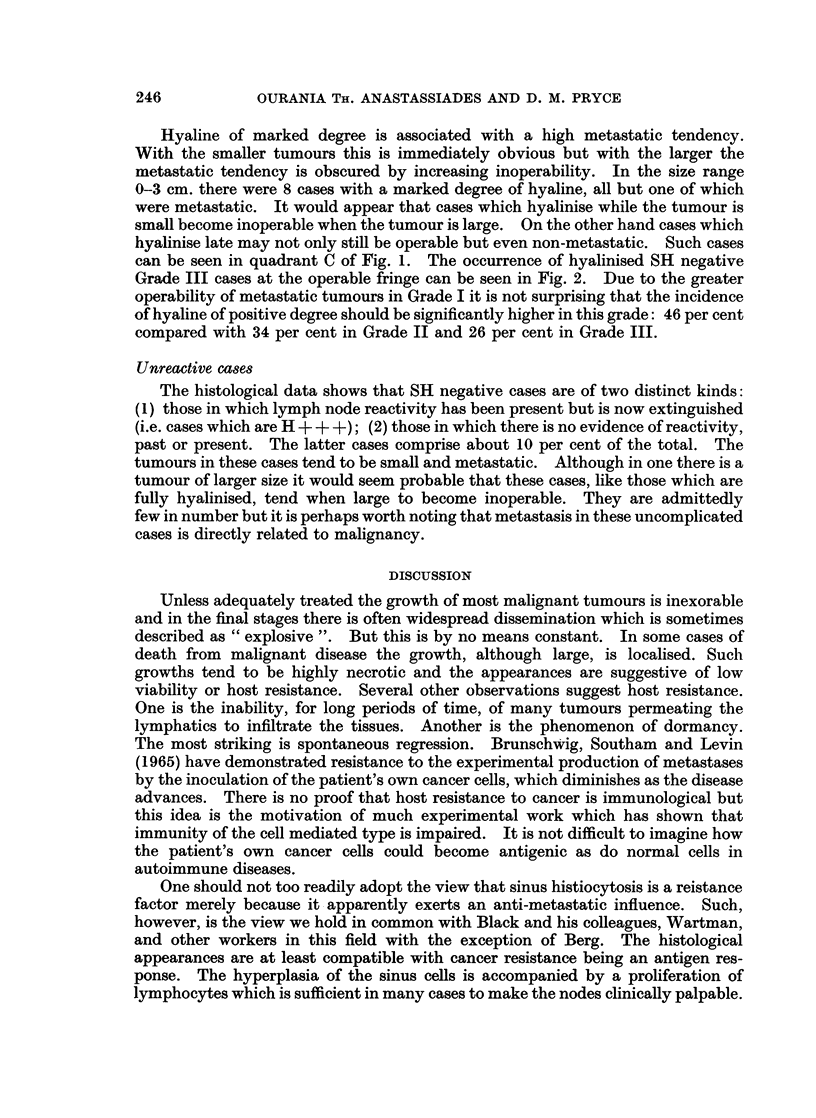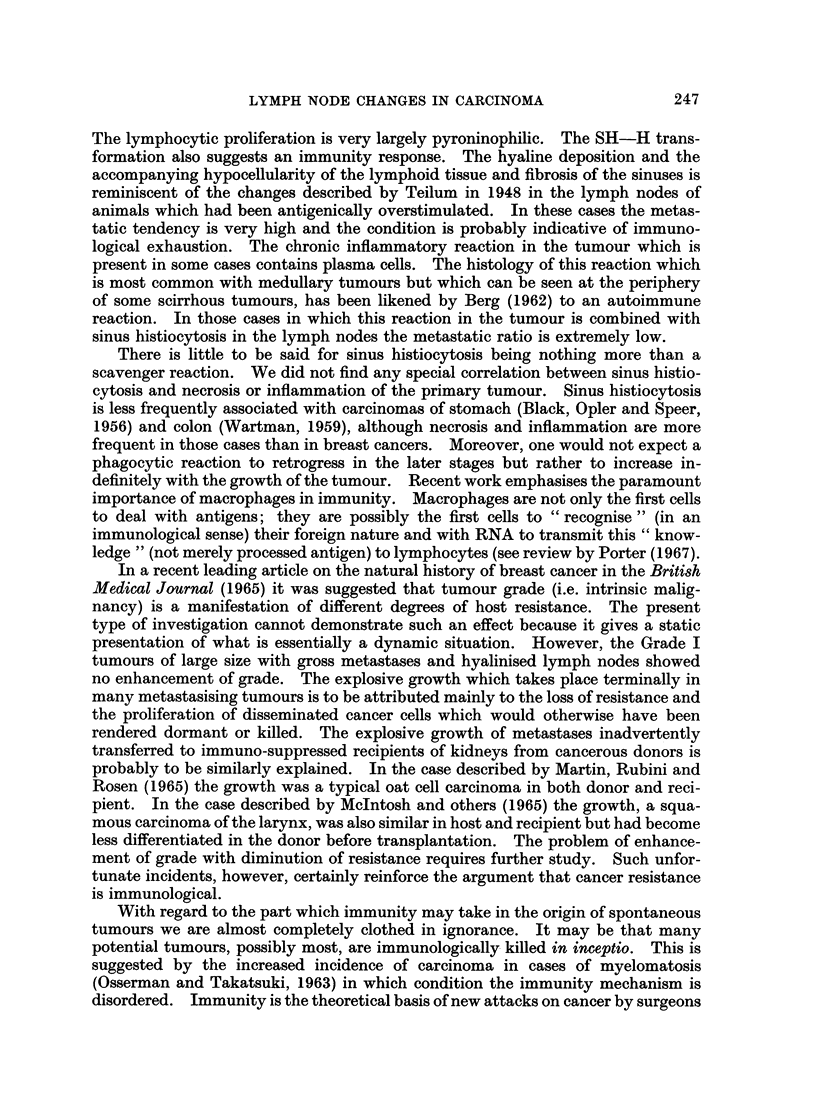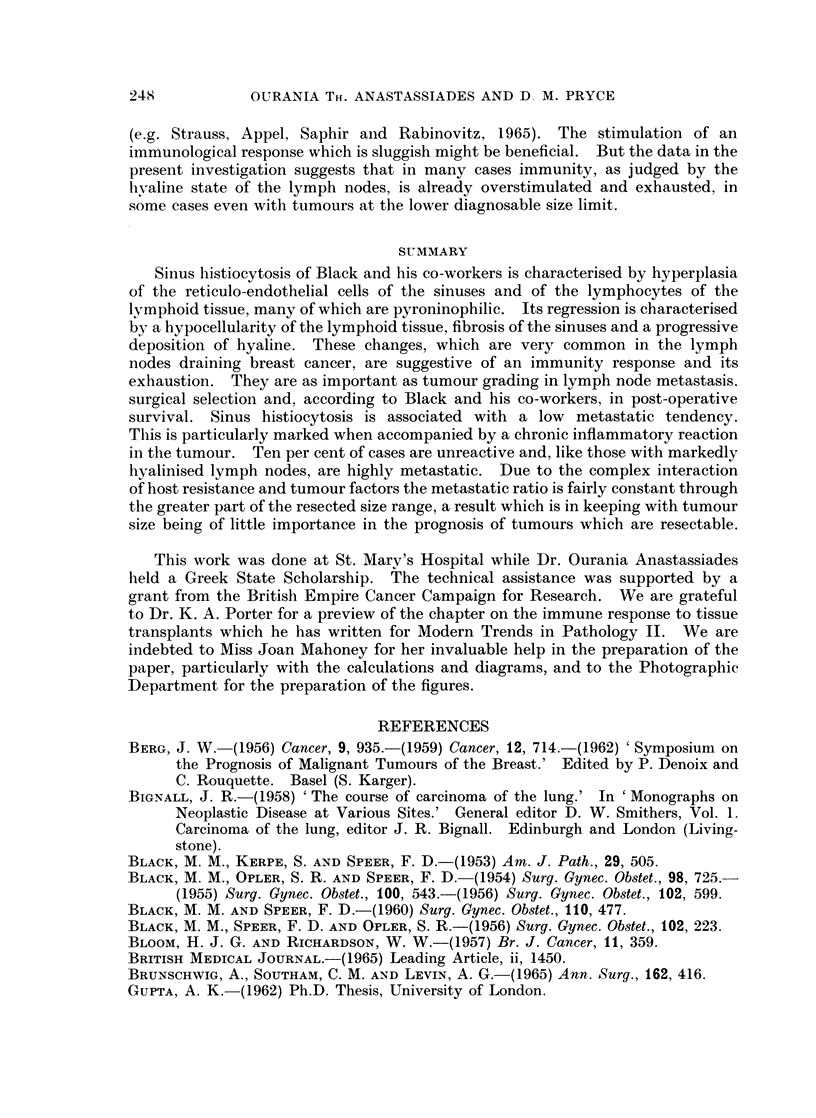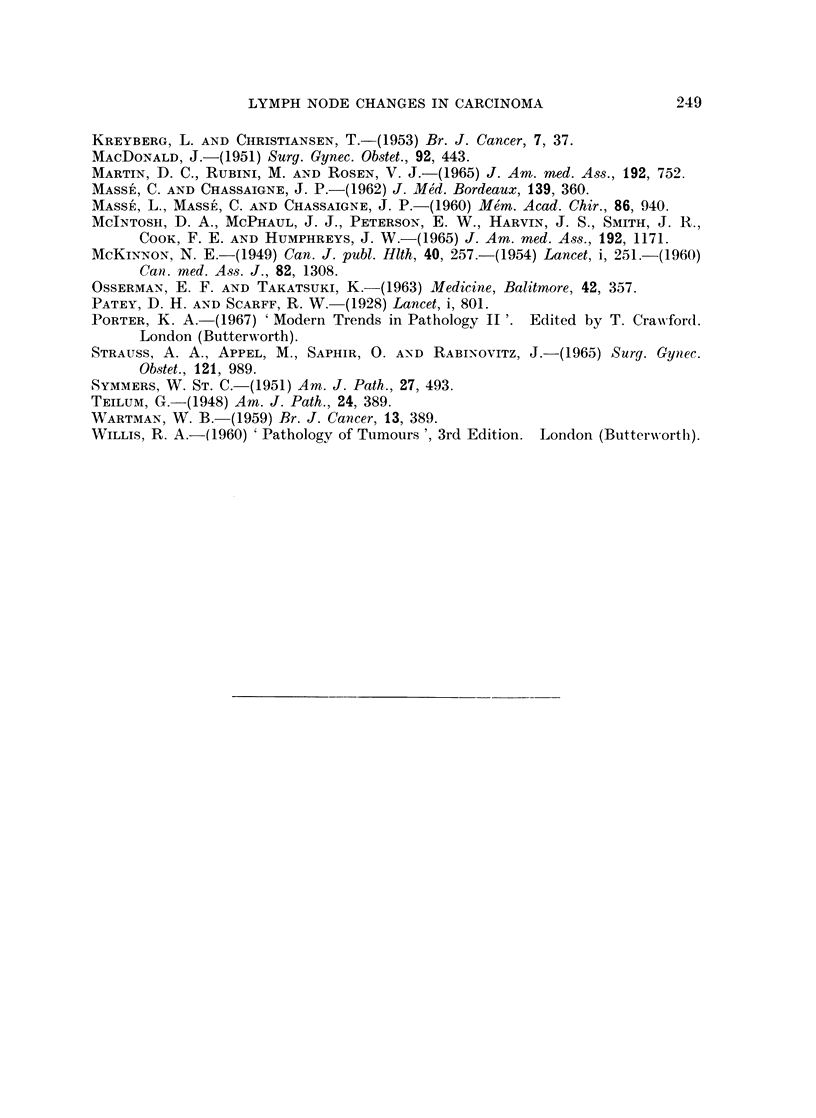# Immunological significance of the morphological changes in lymph nodes draining breast cancer.

**DOI:** 10.1038/bjc.1966.30

**Published:** 1966-06

**Authors:** O. T. Anastassiades, D. M. Pryce


### IMMUNOLOGICAL SIGNIFICANCE OF THE MORPHOLOGICAL CHANGES IN LYMPH NODES DRAINING BREAST CANCER

OURANIA TH. ANASTASSIADES* AND D. M. PRYCE

*From the Department of Pathology, St. Mary's Hospital, London, W.2*

Received for publication April 1, 1966

A VAST amount of work has been done on the morphological grading of malignant tumours and its correlation with post-operative survival. The value of such work has been doubted by some authors, e.g. Willis (1960) who considers that tumour morphology can give only a general impression of malignancy. The prognostic use of grading, however, is one of the justifiable aims of pathology, and the results obtained by Bloom and Richardson (1957) in breast cancer using the method devised by Scarff (Patey and Scarff, 1928) are encouraging. They are especially impressive when grading is taken together with staging. These results, however, are statistical and not sufficiently constant to be confidently used in individual cases, possibly because they express only one aspect of the problem.

More and more it is being recognised that the course of the cancerous process is not simply dependent upon the intrinsic malignancy of the growth but is the resultant of a complicated interplay between opposing groups of factors: those which are intrinsic in the tumour, associated with different degrees of aggression, and those in the host associated with different degrees of resistance. On the basis of this assumption it would be possible for a tumour of high malignancy to be associated with high resistance and to simulate in its behaviour a tumour of low malignancy. This conception has been given by MacDonald (1951) the designation " biological predeterminism ".

Such a defence mechanism against tumour growth has been put forward by Black, Kerpe and Speer (1953), Black, Opler and Speer (1954) and Black and Speer (1960) who studied sinus histiocytosis† in lymph nodes draining various neoplasms, especially those of the breast, and who found a close relationship between this condition and long survival after resection. These authors found that sinus histiocytosis was more frequent in cases without metastases. They also found that in metastatic cases survival was better when sinus histiocytosis accompanied the metastatic growth. These observations prompted Black and his associates to think that the low metastatic ratio and good prognosis were both directly due to sinus histiocytosis. The observations have been confirmed by many workers, including Berg (1956, 1959), Wartman (1959), Massé, Massé and Chassaigne (1960) and Massé and Chassaigne (1962). Berg hotly denies that sinus histiocytosis is due to host resistance but his argument seems obscure. Wartman regards sinus histiocytosis as a resistance factor but in his series of cases he found it most frequently with Grade I tumours, with which of course survival tends to be good.

* Present address: Sina 23, Athens 144, Greece.

† More familiarly known to British pathologists as " sinus catarrh ". See Wartman (1959) for other synonyms and a very comprehensive review of the literature.

We did not find any such difference ourselves, neither did other workers, including Black and his colleagues and Berg.

Another common change in lymph nodes draining breast cancer is the deposition of hyaline, the histology of which was described by Symmers (1951). This and the less common sarcoid appearance was regarded by Black *et al.* (1953) as an extreme expression of sinus histiocytosis. Wartman, on the other hand, considered that the two conditions were unrelated. We decided to estimate the degree of hyaline quantitatively and found an inverse relationship between the two conditions which we think significant.

Black *et al.* possibly overreach themselves when they claim that five year survival after resection can be confidently predicted in cases with marked sinus histiocytosis irrespective of grade or metastasis (Black, Opler and Speer, 1954, 1955; Black, Speer and Opler, 1956). They have, however, re-opened a research domain for investigation which seems important: the reactivity of the reticular tissue of the host against cancer. We had no survival data but we considered that a study of lymph nodes draining breast cancer would be worth while because in relation to metastasis it is easy to show that grading alone is quite inadequate.

### MATERIAL AND METHODS

The study was based on 116 cases of carcinoma of the breast from the files of the Pathology Department of St. Mary's Hospital. The cases were consecutive except for the exclusion of those without detailed data regarding the size of the primary tumour and/or adequate material for study. Fifty-nine of the 116 cases had metastases in the regional lymph nodes. In 11 cases, however, the available lymph nodes were entirely replaced by growth and in 5 cases the tumours were ungradable. In 100 cases the tumours were gradable and the lymphoid tissue could be studied. The tumours were graded according to the method devised by Scarff, subdivided according to fibre content and a note made of the degree of inflammatory infiltration when this was present. In the lymph nodes the presence or absence of sinus histiocytosis was noted and its degree estimated. Sinus histiocytosis in some degree was very common and was completely absent in only a small proportion of cases. Not all lymph nodes in any given case showed the same degree of sinus histiocytosis. Lymph nodes showing moderate and marked sinus histiocytosis could often be found in the same patient. The degree varied, sometimes even in different parts of the same node. These observations agree with those of other workers who, like ourselves, found considerable difficulty in the quantitative estimation of the condition. We finally decided, like previous workers, to classify cases according to the greatest reaction present. We did not use the grading 0–4+ of Black *et al.* but the simpler one of Wartman, in which cases are divided into two main groups according as they show no sinus histiocytosis or only the slightest trace (" SH negative " cases) and cases in which it is present in moderate and marked degree (" SH positive " cases).

The quantitative estimation of hyaline was done in the same general way as for sinus histiocytosis, i.e. according to the most marked degree in which it was present. We again used two main categories: cases showing no hyaline (H−) or only a trace (H+) (" negative " cases); and those showing moderate (H++) or marked (H+++) hyaline (" positive " cases). Sections from all blocks were studied by the H. & E. technique and Unna Pappenheim. The influence of sinus histiocytosis and hyaline on the behaviour of tumours was studied by noting the effect on the metastatic ratio.

### RESULTS

The data is presented in three scatter diagrams. In each figure the cases are distributed about a centimeter scale, the metastatic cases above and the non-metastatic cases below the scale. Seven cases with only minute metastases are placed on the scale itself. Fig. 1 gives the data for the whole collection of 116

FIG 1.—Distribution of the whole collection of tumours according to size and metastasis. Except for the very largest tumours the metastatic ratio is approximately constant at about 50 per cent.

cases, Fig. 2 the resected size range of the 111 scirrhous or non-scirrhous tumours which were gradable, and Fig. 3 the data for the 100 gradable tumours in which the lymphoid tissue could be studied. A key to the symbols denoting grade and fibre content of the tumours and the degree of sinus histiocytosis and hyaline change in the lymph nodes is given in Fig. 1. The presence of a cellular reaction in the tumour is indicated by an exaggeration of the lower tag. Three degrees are indicated, the greatest degree being complete blackening of the tag. This condition, however, will be more closely studied in a subsequent paper.

*Intrinsic malignancy*

Scarff grading of breast cancer is based on morphological criteria relating to rate of tumour growth so that within each grade size gives a rough idea of time. In doing this, however, it should be noted that scirrhous tumours are shrunken and correspond to non-scirrhous tumours of a larger size. Fig. 2 shows that Grade I tumours are resected to a greater size limit than Grade II, and Grade II tumours to a greater size limit than Grade III, and that within each grade non-scirrhous tumours are resected to a greater size limit than scirrhous tumours. These size differences, however, inadequately express differences in time because the time scales of the three grades are so different. Grade I tumours are very slow-growing and much older than tumours of the other two grades. As Kreyberg and Christiansen (1953) point out, their high metastatic ratio (equal even when small to that of Grade III tumours) is not due to aggressiveness but to the great availability of time for metastases to form. Their malignancy is so low that many remain

FIG. 2.—Resected size range of the three grades showing the smaller range of scirrhous tumours. Although the modal size is similar the retrospective duration to this point (which is related to the lower diagnosable size limit) will of course be far longer with Grade I than with Grade III.

resectable even when after years of slow growth they have attained a large size. The aggressiveness of Grade III tumours is associated with rapid growth, and when metastatic, with rapidly increasing inoperability. The great difference between the types has been repeatedly emphasised by McKinnon (1949, 1954, 1960) in connection with mortality statistics. The inoperability rate quoted by Kreyberg and Christiansen was about 25 per cent; ours would be at least as great because the population from which the material was derived had not been submitted to intensive cancer propaganda.

The composite scatter diagram (Fig. 1) shows a swelling (above and below the size scale) which is an additive effect at the modal size common to the three grades. It also shows the differential effects of surgical selection. With increase in tumour size the metastatic ratio of Grade I rises because the tumours of this grade remain operable though metastatic. With Grade III the metastatic ratio falls with increase in tumour size, because the metastatic cases tend to become inoperable. The metastatic ratio of Grade II remains about the same. As the number of cases in the three grades is similar, the metastatic ratio of the whole collection is approximately constant (at about 50 per cent) through the greater part of the resected size range. With the largest tumours there is a slight rise because these are mostly Grade I. A similar scatter diagram, but over a greater size range, was obtained by Gupta (1962) with peripheral tumours of the lung. These find-

FIG. 3.—Distribution of SH positive and negative cases according to grade, size, metastasis, tumour type and hyaline change.

ings are in keeping with the observation of many workers that size is of little significance in post-operative survival and very largely an explanation, because survival is so closely related to metastasis.

Surgical selection, however, is not entirely due to differences in intrinsic malignancy as will be seen from an examination of the grade admixture in the tumour groups when Fig. 1 is divided into quadrants by an arbitrary line to the right of 3 cm.. In spite of the inoperability of many cases the grade admixture of the tumours in quadrants A, B and C combined is similar to that in D, from which they are derived: Grade I 28 per cent in both D and ABC; Grade II 39 per cent in D and 38 per cent in ABC; Grade III 33 per cent in D and 35 per cent in ABC. The grades in A, B and C, however, are unevenly distributed. Surgical exclusion of highly malignant tumours is most evident in quadrant B in which there is a paucity of Grade III. The greatest contrast is between quadrant A (containing the smaller metastatic tumours) and quadrant C (containing the larger non-metastatic tumours). Large tumours which are non-metastatic in spite of their size have been presumed to be of low malignancy, but quadrant C is the one containing the highest proportion of Grade III tumours. Corresponding tumours in the lung have been aptly described by Bignall (1958) as indolent. The metastatic indolence of most of these tumours, however, is not intrinsic and can only be explained on the assumption of host resistance.

*Histological changes in the lymph nodes*

The reactive changes of sinus histiocytosis may cause obvious enlargement of lymph nodes, especially of those nearest the tumour. The opacity of the cut surface, especially when hyaline is also present, may easily be mistaken for growth. When sinus histiocytosis is marked the cells filling the sinuses are nearly always swollen with round or oval nuclei and abundant cytoplasm which is only lightly eosinophilic. The hyperplasia is most marked in the sinuses of the medulla and is accompanied by an increased cellularity of the lymphoid tissue which also is more marked in the medulla. The lymphocytic proliferation is largely pyroninophilic; at least half the lymphocytes in the medullary cords show this change. On the other hand, in cases with only moderate sinus histiocytosis the proliferated sinus cells show degenerative changes. They are often smaller, with cytoplasm which is more eosinophilic, and nuclei which are elongated and hyperchromatic. These changes are accompanied by a decrease in the number of lymphocytes especially in the cortex. Cases in which sinus histiocytosis is slight or absent do not form a homogeneous group. The appearances in some cases suggest the final stages in the complete regression of the condition. The lymphoid tissue in these shows a marked hypocellularity and the sinuses are fibrosed. In other SH negative cases, however, the sinuses are inconspicuous, or show only an oedematous distension due to obstruction.

In those cases in which the condition of sinus histiocytosis shows evidence of regression there is an accompanying deposition of hyaline. When present in slight amount the hyaline change is restricted to the small vessels of the cortex. When more abundant it is present also in the larger vessels and/or in the lymphoid tissue. Hyaline is minimal in lymph nodes with marked sinus histiocytosis, more evident in nodes with only moderate sinus histiocytosis and most marked in hypocellular lymph nodes with fibrosed sinuses. In cases showing little or no evidence of past or present sinus histiocytosis there was no hyaline.

*Cases with active sinus histiocytosis*

There is general agreement among workers in this field that cases with sinus histiocytosis have a lower metastatic ratio than cases in which it is slight or absent. The present results confirm this not only for the collection as a whole but for the separate grades (Fig. 3). The metastatic ratio of the 67 " SH positive " cases is 33 per cent and that of the 38 " SH negative " cases 68 per cent. In Grade I the respective figures are 45 per cent and 87 per cent; in Grade II 33 per cent and 59 per cent and in Grade III 26 per cent and 73 per cent. Wartman (1959) found a greater frequency of SH in Grade I but we were not able to confirm this. The frequency of SH in the whole collection was 64 per cent. The frequency in Grade I was 71 per cent; in Grade II 55 per cent; and in Grade III 68 per cent.

These figures, however, must be seen against the background of surgical selection. Since sinus histiocytosis exerts a protective influence against metastasis one would expect it to be positively selected in the Darwinian sense and therefore in surgical material to show an increased incidence with increase in tumour size, due to the progressive elimination of unprotected cases with more frequent metastases. Instead of the expected rise, however, there is a slight but definite fall: in the 75 smaller tumours (up to 3 cm.) the incidence of SH was 67 per cent, while in the 30 larger tumours the incidence was 57 per cent. There is also a slight fall in the frequency of SH in scirrhous tumours (which are older) compared with non-scirrhous tumours of the same size range: in tumours below 3 cm., an overall reduction of 21 per cent. This fall in frequency with the passage of time instead of the expected rise appears to be due to involution: to " SH positive " cases becoming " SH negative " cases. The histological data supports this view.

*Hyaline positive cases*

The histological data shows that *pari passu* with the involution of sinus histiocytosis there is an increase in hyaline. This inverse relationship between the two processes is shown quantitatively in Table I.

TABLE I.—*Incidence of Sinus Histiocytosis (SH) and Hyaline Change in Lymph Nodes*

| | Hyaline | | | |
|---|---|---|---|---|
| | " Negative " | | " Positive " | |
| SH | − | + | ++ | +++ |
| 25++ . | 12 | 12 | 1 | 0 |
| 42+ . | 7 | 20 | 13 | 2 |
| 38− . | 11 | 7 | 9 | 11 |
| 105 . | 69 | | 23 | 13 |

In the 25 SH++ cases (those with marked sinus histiocytosis) there was no case with marked hyaline and only one with moderate hyaline. In the 23 H++ cases (those with hyaline of moderate degree) 14 also had SH but in only one case was this of marked degree. Again, in the 13 H+++ cases (those with marked hyaline) there were only 2 with SH and in both instances this was only moderate. It will be seen from the diagrams that the transition from the stage of being SH positive, through that of being SH and H positive, to being only H positive may occur at any point in the resected size range and is fully completed in some cases even with tumours of small size.

Hyaline of marked degree is associated with a high metastatic tendency. With the smaller tumours this is immediately obvious but with the larger the metastatic tendency is obscured by increasing inoperability. In the size range 0–3 cm. there were 8 cases with a marked degree of hyaline, all but one of which were metastatic. It would appear that cases which hyalinise while the tumour is small become inoperable when the tumour is large. On the other hand cases which hyalinise late may not only still be operable but even non-metastatic. Such cases can be seen in quadrant C of Fig. 1. The occurrence of hyalinised SH negative Grade III cases at the operable fringe can be seen in Fig. 2. Due to the greater operability of metastatic tumours in Grade I it is not surprising that the incidence of hyaline of positive degree should be significantly higher in this grade: 46 per cent compared with 34 per cent in Grade II and 26 per cent in Grade III.

*Unreactive cases*

The histological data shows that SH negative cases are of two distinct kinds: (1) those in which lymph node reactivity has been present but is now extinguished (i.e. cases which are H+++); (2) those in which there is no evidence of reactivity, past or present. The latter cases comprise about 10 per cent of the total. The tumours in these cases tend to be small and metastatic. Although in one there is a tumour of larger size it would seem probable that these cases, like those which are fully hyalinised, tend when large to become inoperable. They are admittedly few in number but it is perhaps worth noting that metastasis in these uncomplicated cases is directly related to malignancy.

### DISCUSSION

Unless adequately treated the growth of most malignant tumours is inexorable and in the final stages there is often widespread dissemination which is sometimes described as " explosive ". But this is by no means constant. In some cases of death from malignant disease the growth, although large, is localised. Such growths tend to be highly necrotic and the appearances are suggestive of low viability or host resistance. Several other observations suggest host resistance. One is the inability, for long periods of time, of many tumours permeating the lymphatics to infiltrate the tissues. Another is the phenomenon of dormancy. The most striking is spontaneous regression. Brunschwig, Southam and Levin (1965) have demonstrated resistance to the experimental production of metastases by the inoculation of the patient's own cancer cells, which diminishes as the disease advances. There is no proof that host resistance to cancer is immunological but this idea is the motivation of much experimental work which has shown that immunity of the cell mediated type is impaired. It is not difficult to imagine how the patient's own cancer cells could become antigenic as do normal cells in autoimmune diseases.

One should not too readily adopt the view that sinus histiocytosis is a reistance factor merely because it apparently exerts an anti-metastatic influence. Such, however, is the view we hold in common with Black and his colleagues, Wartman, and other workers in this field with the exception of Berg. The histological appearances are at least compatible with cancer resistance being an antigen response. The hyperplasia of the sinus cells is accompanied by a proliferation of lymphocytes which is sufficient in many cases to make the nodes clinically palpable.

The lymphocytic proliferation is very largely pyroninophilic. The SH—H transformation also suggests an immunity response. The hyaline deposition and the accompanying hypocellularity of the lymphoid tissue and fibrosis of the sinuses is reminiscent of the changes described by Teilum in 1948 in the lymph nodes of animals which had been antigenically overstimulated. In these cases the metastatic tendency is very high and the condition is probably indicative of immunological exhaustion. The chronic inflammatory reaction in the tumour which is present in some cases contains plasma cells. The histology of this reaction which is most common with medullary tumours but which can be seen at the periphery of some scirrhous tumours, has been likened by Berg (1962) to an autoimmune reaction. In those cases in which this reaction in the tumour is combined with sinus histiocytosis in the lymph nodes the metastatic ratio is extremely low.

There is little to be said for sinus histiocytosis being nothing more than a scavenger reaction. We did not find any special correlation between sinus histiocytosis and necrosis or inflammation of the primary tumour. Sinus histiocytosis is less frequently associated with carcinomas of stomach (Black, Opler and Speer, 1956) and colon (Wartman, 1959), although necrosis and inflammation are more frequent in those cases than in breast cancers. Moreover, one would not expect a phagocytic reaction to retrogress in the later stages but rather to increase indefinitely with the growth of the tumour. Recent work emphasises the paramount importance of macrophages in immunity. Macrophages are not only the first cells to deal with antigens; they are possibly the first cells to " recognise " (in an immunological sense) their foreign nature and with RNA to transmit this " knowledge " (not merely processed antigen) to lymphocytes (see review by Porter (1967).

In a recent leading article on the natural history of breast cancer in the *British Medical Journal* (1965) it was suggested that tumour grade (i.e. intrinsic malignancy) is a manifestation of different degrees of host resistance. The present type of investigation cannot demonstrate such an effect because it gives a static presentation of what is essentially a dynamic situation. However, the Grade I tumours of large size with gross metastases and hyalinised lymph nodes showed no enhancement of grade. The explosive growth which takes place terminally in many metastasising tumours is to be attributed mainly to the loss of resistance and the proliferation of disseminated cancer cells which would otherwise have been rendered dormant or killed. The explosive growth of metastases inadvertently transferred to immuno-suppressed recipients of kidneys from cancerous donors is probably to be similarly explained. In the case described by Martin, Rubini and Rosen (1965) the growth was a typical oat cell carcinoma in both donor and recipient. In the case described by McIntosh and others (1965) the growth, a squamous carcinoma of the larynx, was also similar in host and recipient but had become less differentiated in the donor before transplantation. The problem of enhancement of grade with diminution of resistance requires further study. Such unfortunate incidents, however, certainly reinforce the argument that cancer resistance is immunological.

With regard to the part which immunity may take in the origin of spontaneous tumours we are almost completely clothed in ignorance. It may be that many potential tumours, possibly most, are immunologically killed *in inceptio*. This is suggested by the increased incidence of carcinoma in cases of myelomatosis (Osserman and Takatsuki, 1963) in which condition the immunity mechanism is disordered. Immunity is the theoretical basis of new attacks on cancer by surgeons

(e.g. Strauss, Appel, Saphir and Rabinovitz, 1965). The stimulation of an immunological response which is sluggish might be beneficial. But the data in the present investigation suggests that in many cases immunity, as judged by the hyaline state of the lymph nodes, is already overstimulated and exhausted, in some cases even with tumours at the lower diagnosable size limit.

### SUMMARY

Sinus histiocytosis of Black and his co-workers is characterised by hyperplasia of the reticulo-endothelial cells of the sinuses and of the lymphocytes of the lymphoid tissue, many of which are pyroninophilic. Its regression is characterised by a hypocellularity of the lymphoid tissue, fibrosis of the sinuses and a progressive deposition of hyaline. These changes, which are very common in the lymph nodes draining breast cancer, are suggestive of an immunity response and its exhaustion. They are as important as tumour grading in lymph node metastasis, surgical selection and, according to Black and his co-workers, in post-operative survival. Sinus histiocytosis is associated with a low metastatic tendency. This is particularly marked when accompanied by a chronic inflammatory reaction in the tumour. Ten per cent of cases are unreactive and, like those with markedly hyalinised lymph nodes, are highly metastatic. Due to the complex interaction of host resistance and tumour factors the metastatic ratio is fairly constant through the greater part of the resected size range, a result which is in keeping with tumour size being of little importance in the prognosis of tumours which are resectable.

This work was done at St. Mary's Hospital while Dr. Ourania Anastassiades held a Greek State Scholarship. The technical assistance was supported by a grant from the British Empire Cancer Campaign for Research. We are grateful to Dr. K. A. Porter for a preview of the chapter on the immune response to tissue transplants which he has written for Modern Trends in Pathology II. We are indebted to Miss Joan Mahoney for her invaluable help in the preparation of the paper, particularly with the calculations and diagrams, and to the Photographic Department for the preparation of the figures.

### REFERENCES

BERG, J. W.—(1956) *Cancer*, **9**, 935.—(1959) *Cancer*, **12**, 714.—(1962) 'Symposium on the Prognosis of Malignant Tumours of the Breast.' Edited by P. Denoix and C. Rouquette. Basel (S. Karger).

BIGNALL, J. R.—(1958) 'The course of carcinoma of the lung.' In 'Monographs on Neoplastic Disease at Various Sites.' General editor D. W. Smithers, Vol. 1. Carcinoma of the lung, editor J. R. Bignall. Edinburgh and London (Livingstone).

BLACK, M. M., KERPE, S. AND SPEER, F. D.—(1953) *Am. J. Path.*, **29**, 505.

BLACK, M. M., OPLER, S. R. AND SPEER, F. D.—(1954) *Surg. Gynec. Obstet.*, **98**, 725.—(1955) *Surg. Gynec. Obstet.*, **100**, 543.—(1956) *Surg. Gynec. Obstet.*, **102**, 599.

BLACK, M. M. AND SPEER, F. D.—(1960) *Surg. Gynec. Obstet.*, **110**, 477.

BLACK, M. M., SPEER, F. D. AND OPLER, S. R.—(1956) *Surg. Gynec. Obstet.*, **102**, 223.

BLOOM, H. J. G. AND RICHARDSON, W. W.—(1957) *Br. J. Cancer*, **11**, 359.

BRITISH MEDICAL JOURNAL.—(1965) Leading Article, ii, 1450.

BRUNSCHWIG, A., SOUTHAM, C. M. AND LEVIN, A. G.—(1965) *Ann. Surg.*, **162**, 416.

GUPTA, A. K.—(1962) Ph.D. Thesis, University of London.

KREYBERG, L. AND CHRISTIANSEN, T.—(1953) *Br. J. Cancer*, **7**, 37.
MACDONALD, J.—(1951) *Surg. Gynec. Obstet.*, **92**, 443.
MARTIN, D. C., RUBINI, M. AND ROSEN, V. J.—(1965) *J. Am. med. Ass.*, **192**, 752.
MASSÉ, C. AND CHASSAIGNE, J. P.—(1962) *J. Méd. Bordeaux*, **139**, 360.
MASSÉ, L., MASSÉ, C. AND CHASSAIGNE, J. P.—(1960) *Mém. Acad. Chir.*, **86**, 940.
MCINTOSH, D. A., MCPHAUL, J. J., PETERSON, E. W., HARVIN, J. S., SMITH, J. R., COOK, F. E. AND HUMPHREYS, J. W.—(1965) *J. Am. med. Ass.*, **192**, 1171.
MCKINNON, N. E.—(1949) *Can. J. publ. Hlth*, **40**, 257.—(1954) *Lancet*, i, 251.—(1960) *Can. med. Ass. J.*, **82**, 1308.
OSSERMAN, E. F. AND TAKATSUKI, K.—(1963) *Medicine, Balitmore*, **42**, 357.
PATEY, D. H. AND SCARFF, R. W.—(1928) *Lancet*, i, 801.
PORTER, K. A.—(1967) ‘ Modern Trends in Pathology II ’. Edited by T. Crawford. London (Butterworth).
STRAUSS, A. A., APPEL, M., SAPHIR, O. AND RABINOVITZ, J.—(1965) *Surg. Gynec. Obstet.*, **121**, 989.
SYMMERS, W. ST. C.—(1951) *Am. J. Path.*, **27**, 493.
TEILUM, G.—(1948) *Am. J. Path.*, **24**, 389.
WARTMAN, W. B.—(1959) *Br. J. Cancer*, **13**, 389.
WILLIS, R. A.—(1960) ‘ Pathology of Tumours ’, 3rd Edition. London (Butterworth).